# Causal relationship between bipolar disorder and inflammatory bowel disease: A bidirectional two-sample mendelian randomization study

**DOI:** 10.3389/fgene.2022.970933

**Published:** 2022-09-20

**Authors:** Zhe Wang, Xinyu Wang, Xushi Zhao, Zhaoliang Hu, Dongwei Sun, Donglei Wu, Yanan Xing

**Affiliations:** ^1^ Department of Surgical Oncology, Department of General Surgery, First Affiliated Hospital, China Medical University, Shenyang, China; ^2^ Department of International Special Medical Center, First Affiliated Hospital, China Medical University, Shenyang, China

**Keywords:** inflammatory bowel disease, mendelian randomization (MR), bipolar disorder, causal relationship, crohn’s disease, ulcerative colitis

## Abstract

**Background:** Growing evidence suggests a bidirectional association between bipolar disorder (BD) and inflammatory bowel disease (IBD); however, observational studies are prone to confounding, making causal inference and directional determination of these associations difficult.

**Methods:** We performed bidirectional two-sample Mendelian randomization (MR) and selected single nucleotide polymorphisms (SNPs) associated with BD and IBD as instrumental variables (IV). SNPs and genetic associations with BD and IBD were obtained from the latest genome-wide association studies (GWAS) in Europeans (BD: cases/controls: 20352/31358; IBD: 12882/21770; Crohn’s disease (CD): 5,956/14927; ulcerative colitis (UC): 6968/20464). The inverse-variance-weighted method was the major method used in MR analyses. MR-Egger, weight mode, simple mode, and weighted median were used for quality control.

**Results:** Genetically predicted BD (per log-odds ratio increase) was significantly positively associated with risk of IBD (OR: 1.18, 95% CI: 1.04–1.33), and UC (OR = 1.19, 95% CI: 1.05–1.35), but not CD (OR = 1.18, 95% CI: 0.95–1.48). The validation analysis found that combined OR of IBD, CD, and UC increased per log-OR of BD were 1.16(95% CI: 1.02–1.31), 1.20(95% CI: 0.98–1.48) 1.17(95% CI: 1.02–1.35), respectively. In contrast, no causal relationship was identified between genetically influenced IBD and BD.

**Conclusion:** Our results confirm a causal relationship between BD and IBD, which may influence clinical decisions on the management of BD patients with intestinal symptoms. Although the reverse MR results did not support a causal effect of IBD on BD, the effect of the IBD active period on BD remains to be further investigated.

## Introduction

Inflammatory bowel disease (IBD) causes a high disease burden worldwide and comprises of two major diseases, Crohn’s disease (CD) and ulcerative colitis (UC), both characterized by visible chronic and progressive intestinal inflammation, weight loss, diarrhea, and gastrointestinal bleeding (Ng et al. 2017, Le Berre et al. 2021). Interactions between genetic predisposition and environmental risk factors including poor dietary habits, antibiotic exposure, smoking, major social stressors, and unfavorable lifestyle are thought to be the main pathogenesis of IBD, as they may contribute to improper intestinal immune activation and disrupt the proinflammatory microbiome (Ananthakrishnan 2015, Piovani et al. 2019, Ramos and Papadakis 2019). However, studies have shown that psycho-neuro-endocrine-immune regulation via the brain-gut axis may also lead to abnormal activation of gut immunity and alter pro-inflammatory flora, suggesting a role in IBD pathogenesis (Bonaz and Bernstein 2013, Gracie et al. 2019). Several recent studies have indicated that individuals with mood disorders may be affected by inflammatory changes in the gut, particularly during the manic and psychotic phases of the disease (Severance et al. 2010, Bernstein et al. 2019, Marrie et al. 2019).

Bipolar disorder (BD), a chronic psychiatric disorder characterized by intermittent mania, depression, or mixed mood states, is an important manifestation of mood disorders (Ferrari et al. 2016). Data from a serological and gene expression study suggests that inflammation may be an important pathology in BD patients (Severance et al. 2014). Another study found that sTNF-R1, IL-1Ra, OPG, and IL-6 were significantly altered in the affective state and that they correlated with the severity of affective symptoms in BD patients (Hope et al. 2011). BD has a high heritability (about 70%), and nonpsychiatric comorbidities (including IBD) are prevalent in patients with BD (Vieta et al. 2018, McIntyre et al. 2020). The increased prevalence of BD in patients with IBD has led to a growing number of studies investigating potential associations between IBD and BD (Severance et al. 2014, Bernstein et al. 2019, Nikolova et al. 2022). For instance, in a cross-sectional study involving over 1.5 million people in the UK, people with BD were nearly twice as likely to develop IBD as those without a BD diagnosis (Smith et al. 2013). This was consistent with the Kao et al. observational study of 3590 IBD patients and 14360 controls from a population survey database in Taiwan (Kao et al. 2019). However, another population-based study from Canada found that patients with IBD had lower BD incidence than the general population (Walker et al. 2008). Conclusions from previous observational studies are controversial, and previously described associations may be affected by reverse causality and residual confounders. Therefore, the directional and causal relationships between BD and IBD remain unclear.

Mendelian randomization (MR) is a more convincing causal reasoning method which minimizes the limitations of observational studies (Emdin et al. 2017, Davey Smith et al. 2020). MR uses genetic variations identified through genome-wide association studies (GWAS) as instrumental variables (IVs) to infer causality between outcome and lifetime exposure, which may effectively avoid confounding factors and reverse causality (Emdin et al. 2017, Porcu et al. 2019, Zhao et al. 2019). Thus, the aim of this study was to investigate the potential bidirectional causal relationship between genetically predicted BD and IBD using the latest and most comprehensive GWAS meta-analysis on IBD and BD, implementing a two-sample MR study design.

## Materials and methods

### Study design

**FIGURE 1:**
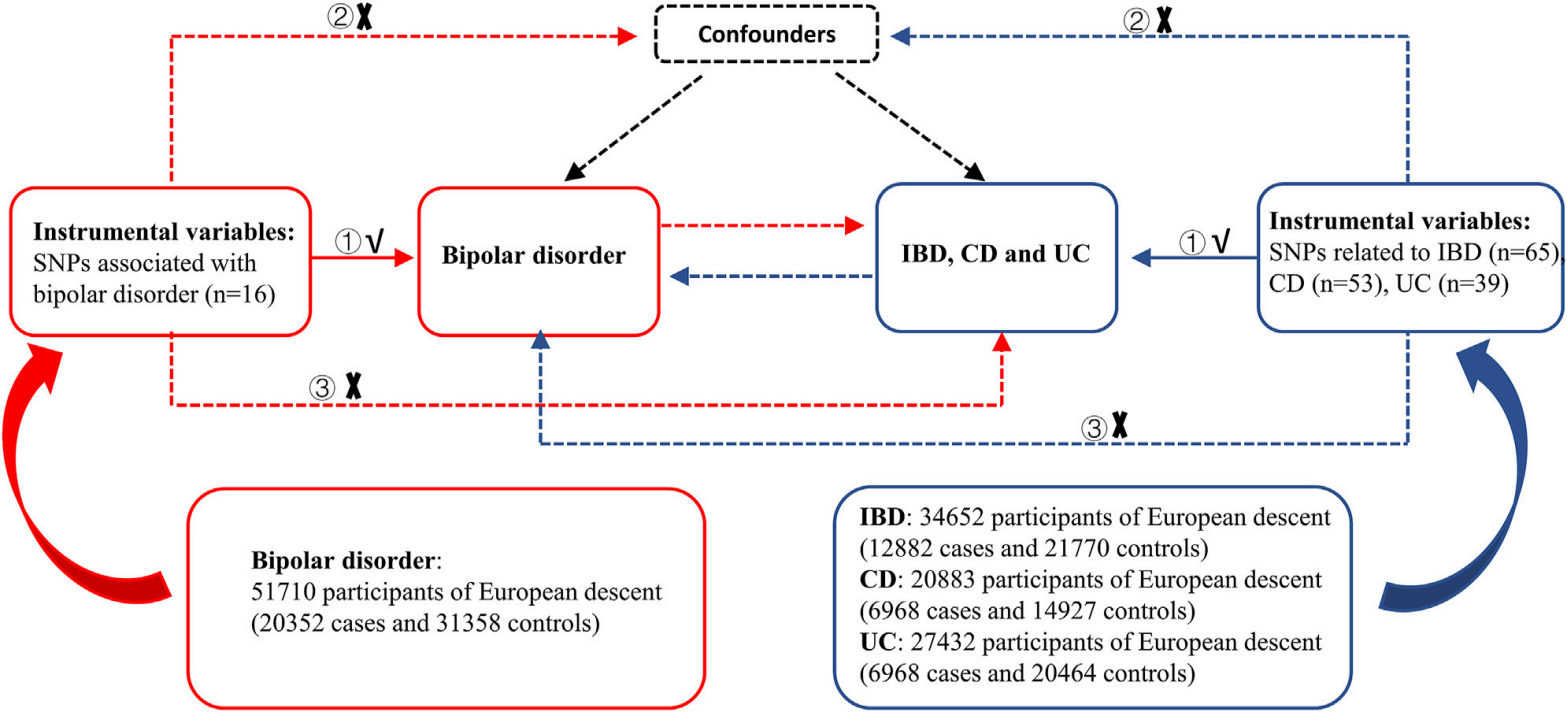
Overview of the study design in this bidirectional MR study. MR analysis depends on three key assumptions: ① IVs should be significantly related to exposure; ② IVs should not be connected to any confounding factors of the exposure-outcome association; ③ IVs affect the outcome only *via* exposure. IBD: inflammatory bowel disease, CD: Crohn’s disease, UC: ulcerative colitis, SNPs: single nucleotide polymorphisms.

A schematic overview of the bidirectional two-sample MR study design and data sources is detailed in Figure 1. The causal relationship of BD with IBD, including UC and CD, was explored using summary-level statistics including the most comprehensive current IBD GWAS of 59957 individuals of European ancestry, then validated using another comprehensive GWAS study from the International Inflammatory Bowel Disease Genetics Consortium (IIBDGC). In reverse MR analysis, summary-level data was extracted from the most extensive current BD-related GWAS, which included 198,882 individuals from 14 countries, to test the association between IBD (including CD and UC) and BD risk. MR depends on three key assumptions: ① IVs should significantly relate to exposure; ② IVs should not connect to any confounding factors of the exposure-outcome association; ③ IVs affect the outcome only via exposure. Our analysis was limited to participants of mostly European ancestry to reduce racial mismatches. Details of the data can be found in the Supplementary Tables.

### Data sources and SNP selection for BD

The latest and most comprehensive published GWAS for BD was used (Stahl et al. 2019), which included 198882 individuals from 14 countries in 32 cohorts including Europe, North America, and Australia. In this study, BD was diagnosed via international consensus criteria DSM-IV or ICD-10 and assessed by trained interviewers, clinically managed checklists, or medical record review using structured diagnostic tools for a lifetime diagnosis of BD. Only patients of European ancestry were including in the present study (20352 cases versus 31358 controls) to reduce bias due to racial mismatch. In most cohorts, controls underwent lifetime psychiatric screening and were randomly selected from the population. GWAS cohort analysis using Plink ‘clumping’ to identify a set of linkage disequilibrium (LD) trimmed found GW AS meta-analyses BD-associated variants (*p* < 0.0001, distance > 500 kilobases (kb) or LD r2 < 0.1) for use in subsequent cohorts analysis. The summary GWAS has undergone strict quality control in cohort analysis, follow-up cohort analysis, genome-wide polygenic risk scores (PRS) analysis, and LD score regression analysis. Logistic regression association tests were performed on BD in each cohort, adjusting for covariates of the seven principal components including age, sex, and genetic ancestry.

We excluded 8 SNPs associated with more than one phenotype (e.g., some SNPs are also associated with schizophrenia) to avoid any potential pleiotropic IVs. After removing pleiotropic SNPs, sixteen independent BD-related loci were identified in this GWAS with genome-wide significance thresholds (*p* < 5 × 10-8), and satisfactory variants were selected to construct instrumental variables.

### Data sources and SNP selection for IBD

A GWAS meta-analysis on IBD was recently conducted by de Lange et al., consisting of 59957 individuals (25042 cases and 34915 controls) of predominantly European ancestry (UC:12366 cases/33609 controls; CD:12194 cases/28072 controls) (de Lange et al. 2017). All included cases were diagnosed by recognized radiological, endoscopic, and histopathological assessments and met clinical diagnostic criteria for IBD. The results of the fixed-effects meta-analysis were further filtered, and sites with strong evidence of heterogeneity (I2>0.90) were discarded. Only sites where all cohorts passed our quality control filters were included in the analysis. Another large GWAS summary of IBD data from Liu et al. was used as a validation analysis (Liu et al. 2015), with a study population arising from Europe, Iran, India or East Asia. To reduce bias caused by racial mismatch, only the population with European ancestry was used as the research object. Mark QC, sample QC, population correlation analysis QC and Genomic inflation factor QC were performed respectively, and all SNPs that did not conform to Hardy-Weinberg equilibrium were eliminated. After quality control (QC) and 1000-genome estimates, adjusted for covariates including smoking status, race, sex, family history, age of disease onset, extraintestinal manifestations and surgery, the number of cases and controls were 12882/21770 for IBD, 5956/14927 for CD, and 6968/20464 for UC, respectively.

For reverse MR analyses, after removing 44 pleiotropic SNPs with more than one phenotype, 65 independent genetic SNPs with *p* values less than 5 × 10−8 were selected from the summary-level GWAS of Liu et al. to construct the IBD genetic instruments (Liu et al. 2015). For CD and UC, 53 and 39 independent genetic SNPs were selected by the same method. At the same time, we also tested the potential causal relationship of de Lange et al IBD GWAS data to BD, details of the significant IBD SNPs of de Lange et al were in Supplementary Table S3.

### Selection of instrumental variables

SNPs were identified at a threshold of genome-wide significance (*p* < 5 × 10-8). Stringent clumping criteria were set to further filter SNPs with low LD (r2 = 0.001 in 10000 Kb windows) and high minor allele frequency (MAF > 0.01). R2 and F statistics were calculated to represent the variance ratio of exposure factors explained by IVs and the association between IVs and risk exposures of interest (Burgess et al. 2011). F-statistics was calculated by the formula: $\left(N-2\right)\times \frac{R^{2}}{1-R^{2}}$ to check for bias due to weak IVs, and it is generally recommended to use an F-statistic threshold >10 for MR analysis (Burgess et al. 2011).

Given the selection of SNPs from a very large GWAS, IVs may have effects on traits other than exposure, such as directly affecting outcomes. If those variants that are more strongly associated with outcome than exposure cannot be excluded from the MR analysis, the MR analysis results may be inaccurate due to the reverse causality between exposure and outcome. Therefore, it is necessary to determine whether the SNP is primarily associated with the exposure of interest rather than the outcome. To clarify the direction of causality for each IV with respect to exposure and outcome, we applied MR Steiger filtering to remove those SNPs that were strongly associated with outcomes (Hemani et al. 2017). Steiger filtering assumes that the IV should explain more exposure variation than the outcome; the direction of the instrument is “TRUE” if the IV meets the criteria, and “FALSE” otherwise. After removing those SNPs with the “FALSE” orientation using Steiger filtering, we proceeded to the next MR analysis.

### Statistical analyses

Inverse variance weighted (IVW) MR was the main method used to estimate the potential bidirectional relationship between BD and IBD since it avoids confounding factors in the absence of horizontal pleiotropy and produces unbiased estimates (Burgess et al. 2013). At the same time, weighted mode, simple mode, weighted median, and MR Egger methods were used for supplementary and substitution analysis (Bowden et al. 2016, Bowden et al. 2018). In MR-Egger regression, the MR-Egger intercept was used to test for directional horizontal pleiotropy effect (Bowden et al. 2015). Cochran’s Q statistic and funnel plots were then used to verify the heterogeneity of the IVW methods and MR-Egger regression. Cochran’s Q-test statistic was used to examine heterogeneity among all SNPs in each database. Finally, the leave-one-out method was used for sensitivity analysis to verify the stability of the results. Leave-one-out analysis was performed by excluding each SNP in turn and applying the IVW method to the remaining SNPs to assess the potential effect of specific variants on the estimates. When Cochran’s Q test suggests that there is heterogeneity in SNPs, leave-one-out analysis can be a good way to verify the stability of MR analysis. All statistical analyses in this study were performed using the TwoSampleMR packages (https://mrcieu.github.io/TwoSampleMR, version 0.5.6) in R (version 4.1.3, www.r-project.org/).

## Results

### The causal effect of BD on IBD

Among the 16 BD-associated variants, one SNP was unavailable in the summary-level GWAS of IBD, UC, and CD. In addition, we excluded five SNPs for IBD, CD, and UC due to ambiguous palindromes. We ultimately included 10 SNPs in the MR analysis as genetic instruments for IBD, CD, and UC. The R2 and the (minimal - maxima) F-statistics (112.62–258.55) indicated that all IVs were suitable for MR analysis. (Supplementary Table S1).

**FIGURE 2:**
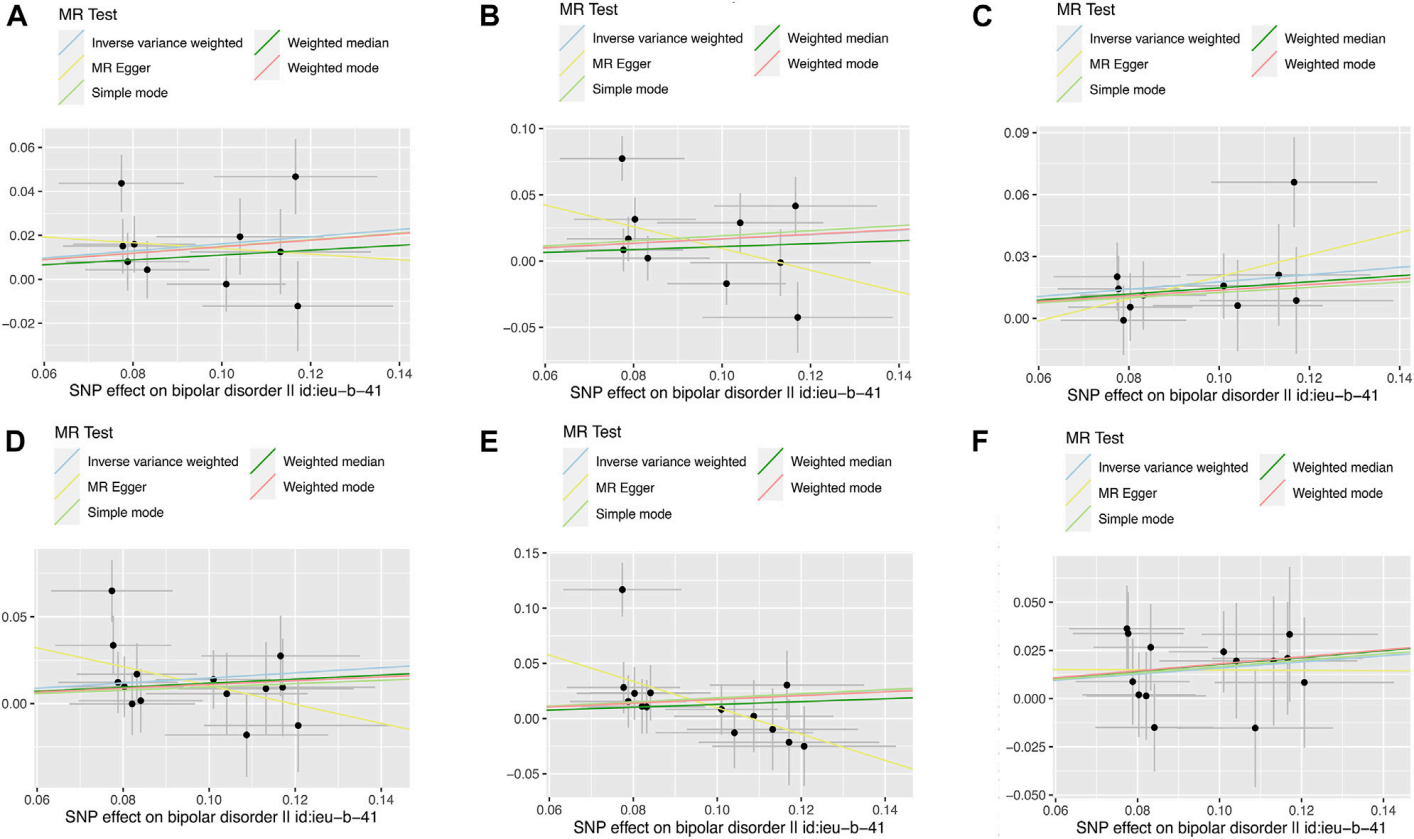
Scatter plots of the relationship between genetically predicted BD on IBD, CD and UC. The x-axes represent the genetic instrument–BD associations and y-axes represent genetic instrument–IBD associations from different outcome databases. Black dots denote the genetic instruments included in the primary MR analyses. The colored lines represent the MR fitting results. The line at each point actually reflects the 95% confidence interval. **(A)** BD on IBD*; **(B)** BD on CD*; **(C)** BD on UC*; **(D)** BD on IBD#; **(E)** BD on CD#; **(F)** BD on UC#. BD: bipolar disorder, IBD: inflammatory bowel disease, CD: Crohn’s disease, UC: ulcerative colitis. * Data from de Lange et al. # Data from Liu et al.

The result of the MR analysis showed that genetically predicted BD significantly positively correlated with IBD. (Table 1). The odds ratio (OR) for IBD with 95% confidence interval (CI) per log-OR increment in BD liability was 1.18 (95% CI: 1.04–1.33; *p* = 0.008) in the IVW model, consistent with the trend of the median weight model, although the median weight model did not reach statistical significance. The scatter diagram is shown in Figure 2 and the forest diagram is shown in Supplementary Figure S1. MR-Egger regression did not reveal a potential horizontal pleiotropy for BD on IBD (egger-intercept = 0.03, *p* = 0.47), which was similar to the conclusion for BD on CD and UC. Cochran’s Q value suggested no notable heterogeneity (Q = 12.82, *p* = 0.12), consistent with the conclusion of the MR analysis shown in the funnel plot (Supplementary Figure S2). Furthermore, as shown in the leave-one-out analysis, no significant association changes were observed after removing any individual variant (Supplementary Figure S3).

**TABLE 1:** Effects of genetically predicted BD on the risk of IBD in the MR analysis.

Exposure	Outcome	No. SNP	Methods	OR (95% CI)	pval	Egger_intercept	p-Egger_intercept
BD	IBD*	10	MR Egger	0.88 (0.41–1.89)	0.749	0.03	0.47
			Weighted median	1.12 (0.97–1.28)	0.119		
			IVW	1.18 (1.04–1.33)	0.008		
			Simple mode	1.16 (0.91–1.48)	0.253		
			Weighted mode	1.16 (0.96–1.40)	0.164		
BD	UC*	10	MR Egger	1.71 (0.79–3.69)	0.211	-0.03	0.38
			Weighted median	1.16 (0.97–1.37)	0.081		
			IVW	1.19 (1.05–1.35)	0.005		
			Simple mode	1.13 (0.90–1.43)	0.312		
			Weighted mode	1.15 (0.90–1.46)	0.289		
BD	CD*	10	MR Egger	0.44 (0.12–1.61)	0.251	0.09	0.17
			Weighted median	1.12 (0.92–1.35)	0.257		
			IVW	1.18 (0.95–1.48)	0.142		
			Simple mode	1.21 (0.90–1.63)	0.243		
			Weighted mode	1.18 (0.86–1.62)	0.319		
BD	IBD#	14	MR Egger	0.58 (0.29–1.17)	0.149	0.06	0.08
			Weighted median	1.12 (0.97–1.30)	0.125		
			IVW	1.16 (1.02–1.30)	0.024		
			Simple mode	1.10 (0.88–1.37)	0.415		
			Weighted mode	1.11 (0.88–1.40)	0.375		
BD	UC#	14	MR Egger	0.99 (0.51–2.39)	0.978	0.02	0.71
			Weighted median	1.19 (0.99–1.43)	0.057		
			IVW	1.17 (1.02–1.35)	0.029		
			Simple mode	1.18 (0.85–1.62)	0.321		
			Weighted mode	1.20 (0.88–1.62)	0.258		
BD	CD#	14	MR Egger	0.30 (0.10–0.89)	0.051	0.13	0.03
			Weighted median	1.13 (0.92–1.39)	0.221		
			IVW	1.20 (0.98–1.48)	0.079		
			Simple mode	1.20 (0.90–1.61)	0.231		
			Weighted mode	1.19 (0.88–1.61)	0.289		

*Data from de Lange et al.

#Data from Liu et al.

BD, on IBD* MR, Egger (Q = 12.82, *p* = 0.12), BD on UC* MR Egger (Q = 5.27, *p* = 0.73), BD on CD* MR Egger (Q = 21.96, *p* = 0.005), BD on IBD# MR, Egger (Q = 11.52, *p* = 0.48), BD on UC# MR Egger (Q = 6.02, *p* = 0.91), BD on CD# MR Egger (Q = 15.23, *p* = 0.22), Q: Cochran’s Q statistics.

BD, bipolar disorder; IBD, inflammatory bowel disease; CD, crohn’s disease; UC, ulcerative colitis; IVW, inverse variance weighted.

Genetic susceptibility of BD had a significant positive correlation with UC (OR = 1.19, 95% CI: 1.05–1.35; *p* = 0.005), but no obvious association was identified for CD (OR = 1.18, 95% CI: 0.95–1.48; *p* = 0.14). (Table 1). Scatter and forest diagrams are shown in Figure 2 and Supplementary Figure S1, respectively. Cochran’s Q test and funnel plot (Supplementary Figure S2) suggested heterogeneity in the CD database (*p* values of Cochran’s Q = 0.005) but not in the UC database (*p* values of Cochran’s Q = 0.73). In addition, the leave-one-out analysis also indicated that the results were stable. (Supplementary Figure S3).

The validation analysis (Table 1) used scatter plots (Figure 2) and forest plots (Supplementary Figure S1) to find MR results consistent with the initial analysis. IVW estimates were analyzed to genetically predict that the combined OR of IBD, CD, and UC increases per log-OR of BD were 1.16(95% CI: 1.02–1.31), 1.20(95% CI: 0.98–1.48), and 1.17 (95% CI: 1.02–1.35), respectively. (Table 1). The Egger’s test showed no potential horizontal pleiotropy except for the relationship between BD and risk of CD. Cochran’s Q test and funnel plot analysis (Supplementary Figure S2) showed significant heterogeneity between BD and CD risk, but no heterogeneity was shown in IBD and UC. The leave-one-out analysis demonstrated the stability of the results (Supplementary Figure S3).

### The causal effect of IBD on BD

In the reverse MR analysis, we utilized 61 variants for IBD, 48 variants for CD, and 35 variants for UC as genetic instruments. A summary and detailed information about the variants for each exposure are presented in Supplementary Table S2.

As shown in Table 2, we observed no causal relationship between genetically determined IBD (including both CD and UC) and BD in the outcome database, with ORs close to 1. The Scatter diagram and forest diagram are shown in Supplementary Figure S4 and Supplementary Figure S5, respectively. The Egger’s test showed no potential horizontal pleiotropy in reverse MR analysis. Cochran’s Q test and funnel plot analysis (Supplementary Figure S6) suggested notable heterogeneity. Therefore, we used a random-effects IVW model to estimate the MR effect size and found no causal relationship between IBD (including CD and UC) and BD. After individual SNPs were deleted, the results remained consistent in the leave-one-out analyses (Supplementary Figure S7). Steiger filtering showed that all genetic IVs used for IBD explained more variance in IBD than in BD in any database (Supplementary Table S2). In addition, we also validated the effect of IBD data on BD from de Lange et al., and the results suggest that there is no casual relationship between IBD and BD (Supplementary Table S4).

**TABLE 2:** Effect of genetically predicted IBD on the risk of BD in the MR analysis.

Exposure	Outcome	No. SNP	Methods	OR (95% CI)	pval	Egger_intercept	p-Egger_intercept
IBD#	BD	61	MR Egger	0.99 (0.90–1.07)	0.74	0.002	0.772
			Weighted median	1.01 (0.97–1.05)	0.64		
			IVW	1.00 (0.97–1.03)	0.88		
			Simple mode	0.98 (0.90–1.07)	0.69		
			Weighted mode	0.99 (0.93–1.05)	0.81		
CD#	BD	48	MR Egger	1.01 (0.94–1.07)	0.96	0.003	0.681
			Weighted median	1.01 (0.97–1.04)	0.55		
			IVW	1.01 (0.99–1.04)	0.34		
			Simple mode	1.04 (0.98–1.10)	0.23		
			Weighted mode	1.02 (0.98–1.06)	0.41		
UC#	BD	35	MR Egger	0.95 (0.87–1.04)	0.29	0.009	0.309
			Weighted median	0.99 (0.95–1.03)	0.66		
			IVW	0.99 (0.96–1.03)	0.73		
			Simple mode	1.01 (0.94–1.10)	0.72		
			Weighted mode	0.99 (0.94–1.05)	0.91		

#Data from Liu et al.

IBD on BD MR Egger (Q = 93.92, *p* = 0.003), CD on BD MR Egger (Q = 87.42, *p* = 0.0002), UC on BD MR Egger (Q = 49.92, *p* = 0.03), Q: Cochran’s Q statistics.

BD, bipolar disorder; IBD, inflammatory bowel disease; CD, Crohn’s disease; UC, ulcerative colitis; IVW, inverse variance weighted.

## Discussion

We tested the potential bidirectional association between BD and IBD and found evidence that genetically predicted BD associates with an increased risk of IBD and UC, with a non-significant trend towards increased risk with CD (Supplementary Table S5). The reverse MR analyses implicated that genetic liability to IBD or any subtype does not significantly associate with BD.

Previous observational studies have shown that BD is positively associated with IBD risk, consistent with our results (Eaton et al. 2010, Smith et al. 2013). However, observational studies on the effect of IBD on BD risk remain controversial (Nikolova et al. 2022). One Canadian study of IBD patients found a lower BD prevalence in IBD patients than in controls, while another cross-sectional study showed that patients with IBD were 2.1 times more likely to develop BD than control subjects (Walker et al. 2008, Kao et al. 2019). These controversial findings may result from methodological limitations and small patient sample sizes. In addition, racial differences and confounding factors may influence the association between BD and IBD. Our MR study was based on the largest available set of GWAS data and restricted the population to those with European ancestry to avoid bias due to small sample size or ethnic differences. Our MR analysis suggests that genetic prediction of IBD is not significantly associated with BD risk, suggesting that the previously observed association may be due to confounding factors or ethnic differences.

Although the biological link between IBD and BD remains unclear, several proposed hypotheses are worth investigating. Recent evidence has shown that patients with BD have significantly higher serological anti-Saccharomyces cerevisiae antibodies (ASCA) levels than non-psychotic patients (Severance et al. 2014). ASCA is commonly used as a predictor of IBD and has significant disease associations with immune reactivity to wheat gluten and bovine casein. However, IBD may accelerate exposure to food antigens to the systemic circulation, which may help explain the elevated levels of gluten and casein antibodies seen in patients with BD (Severance et al. 2014). Another possible hypothesis is that the digestive byproducts of these foods are exorphins which may directly interact with tight junction proteins or undergo epithelial cell transcytosis to potentially affect brain physiology by acting on opioid receptors (Peeters et al. 2001, Lammers et al. 2008, Tripathi et al. 2009). Secondly, many previous studies have suggested that inflammatory cytokines may play a key role in IBD pathogenesis (Neurath 2014, Friedrich et al. 2019), and psychiatric diseases promote intestinal inflammation by regulating the microbiota-gut-brain axis (Osadchiy et al. 2019). Altered mood increases gut permeability, enabling gut bacteria to translocate to peripheral lymphoid organs and trigger innate immune responses (Peppas et al. 2021). Affective disorders can activate the hypothalamic-pituitary-adrenal axis, thereby aggravating chronic inflammation and promoting immune response, consistent with previous observations that BD patients have elevated levels of inflammatory cytokines (Modabbernia et al. 2013, Munkholm et al. 2013, Kostic et al. 2014, Gracie et al. 2019). Activation of the hypothalamic-pituitary-adrenal axis stimulates secretion of corticotropin-releasing factor (CRF), followed by release of adrenocorticotropic hormone (ACTH) from the anterior pituitary. CRF and ACTH increase intestinal permeability by inducing mast cell degranulation and cytokine secretion (Santos et al. 1999, Hill et al. 2013). In addition, BD also stimulates activation of the sympathetic nervous system through the stress response, mediating changes in the autonomic nervous system and increasing catecholamine secretion to exert a pro-inflammatory effect (Farhadi et al. 2005, Luo et al. 2021). A series of inflammatory reactions caused by BD increases intestinal permeability and damages the epithelial barrier, further activating the immune response to disrupt gastrointestinal homeostasis and ultimately lead to IBD.

Two main advantages of our study are worth noting. Observational studies suggest a bidirectional relationship between BD and IBD, but studies present opposing conclusions due to potential confounding factors. A major advantage of this MR study is that we explored the results from a genetic susceptibility perspective, avoiding reverse causality and minimizing residual confounding. Second, we used the largest available resource of exposure GWAS data and the broadest summary-level IBD and BD data from different samples and validated our results across different datasets. Although potential sample overlap cannot be completely avoided, two-sample MR greatly reduces bias due to potential sample overlap between exposures and outcomes. The consistency of the two analyses suggests our results are accurate.

However, some limitations should be acknowledged. First, our study subjects were primarily individuals of European ancestry, which may limit the generality of our findings to other ethnic groups. However, selecting populations of the same ancestry for studies helps avoid genetic differences between races, making our conclusions more convincing. Second, previous studies have shown that patients with active IBD have significantly higher rates of affective disorders than patients with inactive disease (Walker et al. 2008, Kao et al. 2019). While our conclusions do not support a causal effect of IBD on BD, the GWAS data we included only considered the dichotomous diagnosis of IBD, i.e., incidence, but not the course of IBD. Because IBD is characterized by alternating remissions and relapses and its onset is difficult to predict, dissecting the genetic makeup associated with IBD activity remains a challenge. Therefore, due to the lack of GWAS data on the active phase of IBD, we were unable to explore the causal relationship between active IBD and BD using MR methods. Third, in partial negative MR results, Cochran’s Q value suggested significant heterogeneity of IVs. Therefore, we performed further random effect IVW analysis and leave-one-out analysis to support the stability of the results.

Despite the well-established bidirectional relationship between IBD and mental illness, psychotherapy for patients with IBD is currently rarely recommended as an adjunctive treatment to improve quality of life (Lamb et al. 2019). The causal relationship of BD to IBD observed in our study should draw attention to intestinal symptoms in BD patients for more accurate clinical treatment. Affective disorders can lead to chronic inflammation and stress response intensification, so clinicians should improve the IBD suspicion index for BD patients (Marrie et al. 2019). Persistent gastrointestinal symptoms should not be ignored, and antidepressant treatments may need to be tailored for their different effects on bowel habits. Multiple studies have also demonstrated that dietary intervention and probiotic therapy can have a positive impact on BD (Liu et al. 2019, Nikolova et al. 2021). Therefore, people with BD and concomitant lower gastrointestinal disorders should consider using this therapy to obtain maximal benefit.

## Conclusion

Our results confirm a causal relationship between BD and IBD, which may influence clinical decisions on the management of BD patients with intestinal symptoms. Although the reverse MR results did not support a causal effect of IBD on BD, the effect of active IBD on BD remains to be further investigated.

## Data Availability

The original contributions presented in the study are included in the article/Supplementary Material, further inquiries can be directed to the corresponding author.
