# Supplementary figures and images for: Causal relationship between bipolar disorder and inflammatory bowel disease: A bidirectional two-sample mendelian randomization study
Source: Front Genet. 2022 Sep 20;13:970933. doi: 10.3389/fgene.2022.970933 (PMC9531165; doi:10.3389/fgene.2022.970933)

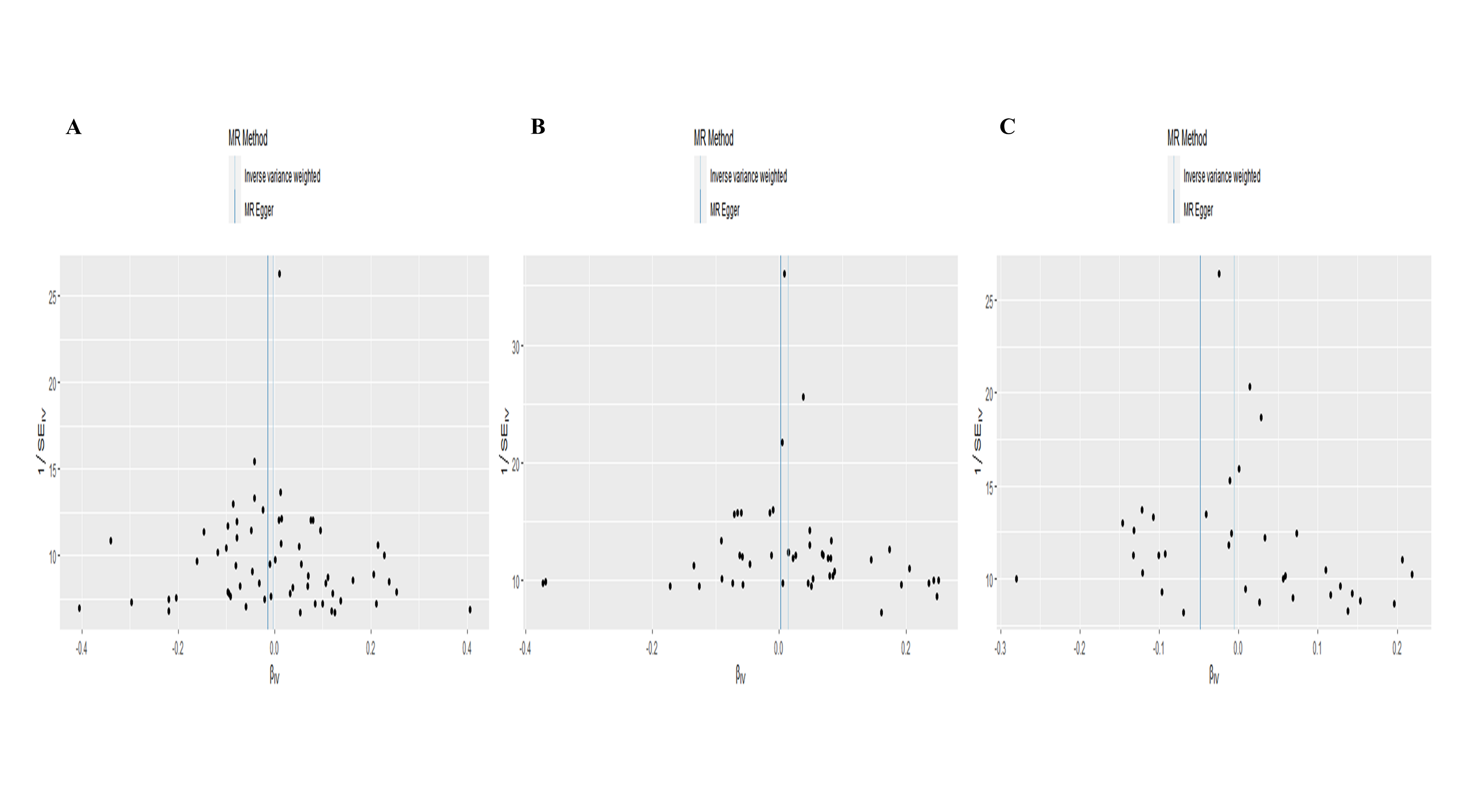

Supplement: Supplementary file 3 [file Image6.TIF]

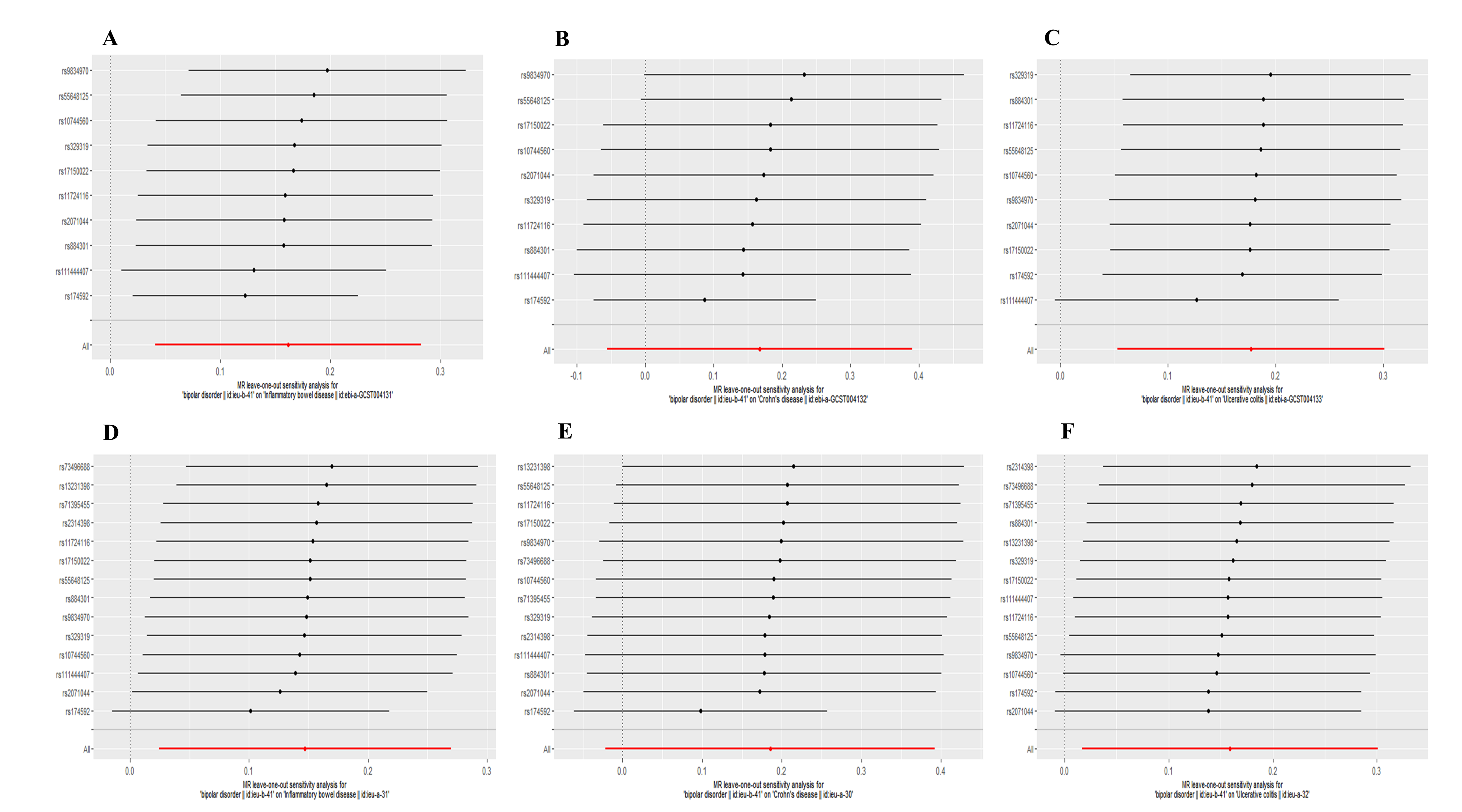

Supplement: Supplementary file 5 [file Image3.TIF]

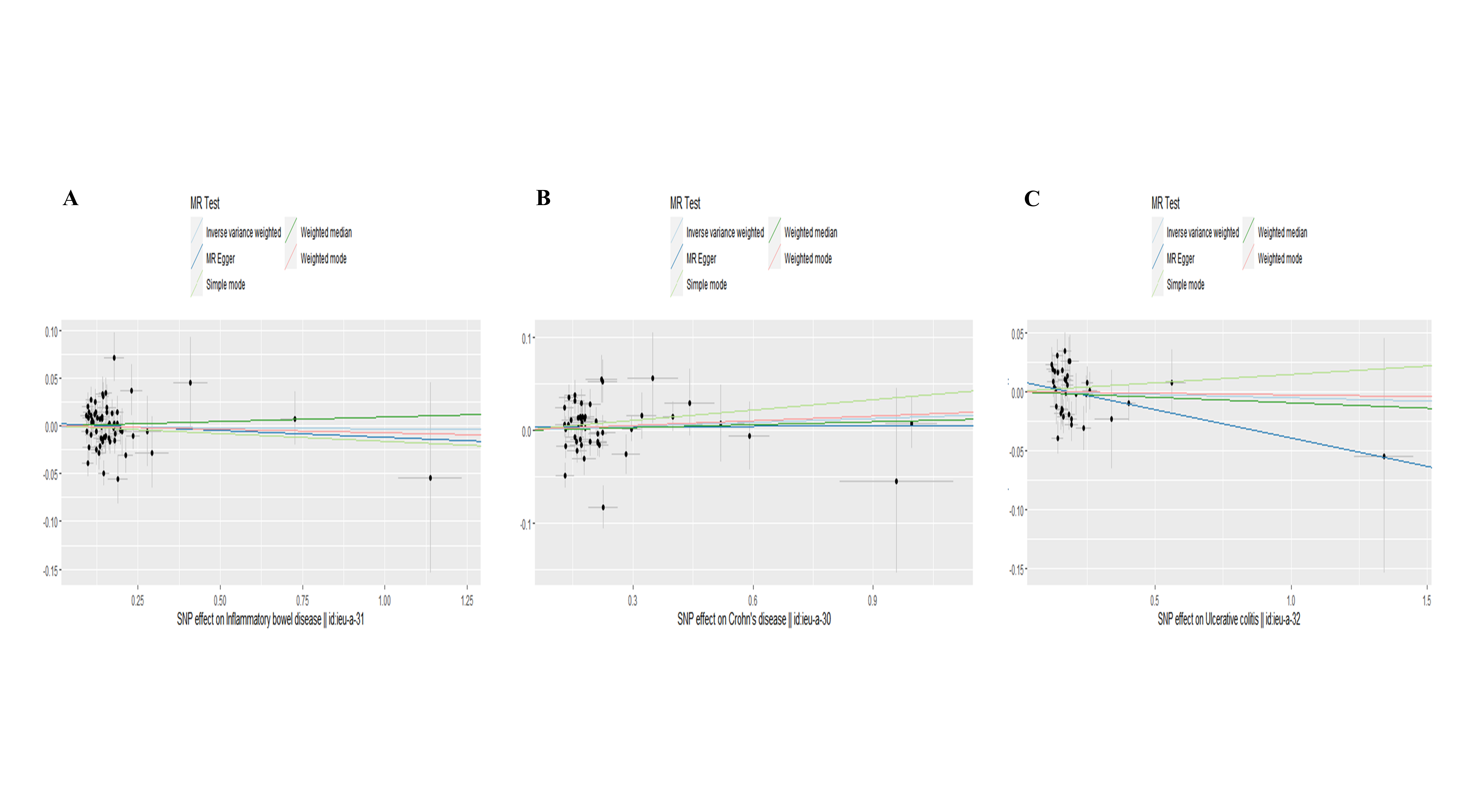

Supplement: Supplementary file 6 [file Image4.TIF]

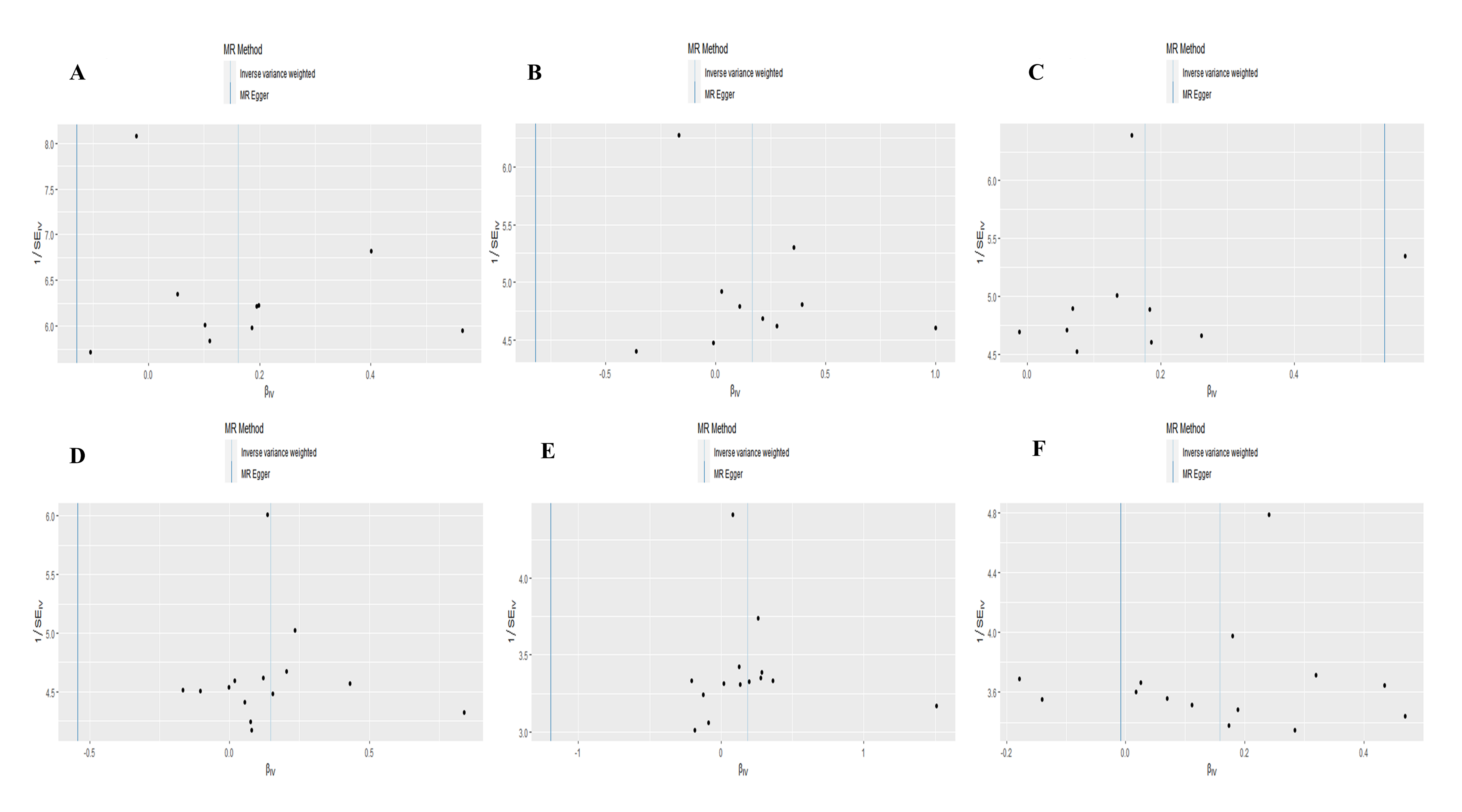

Supplement: Supplementary file 7 [file Image2.TIF]

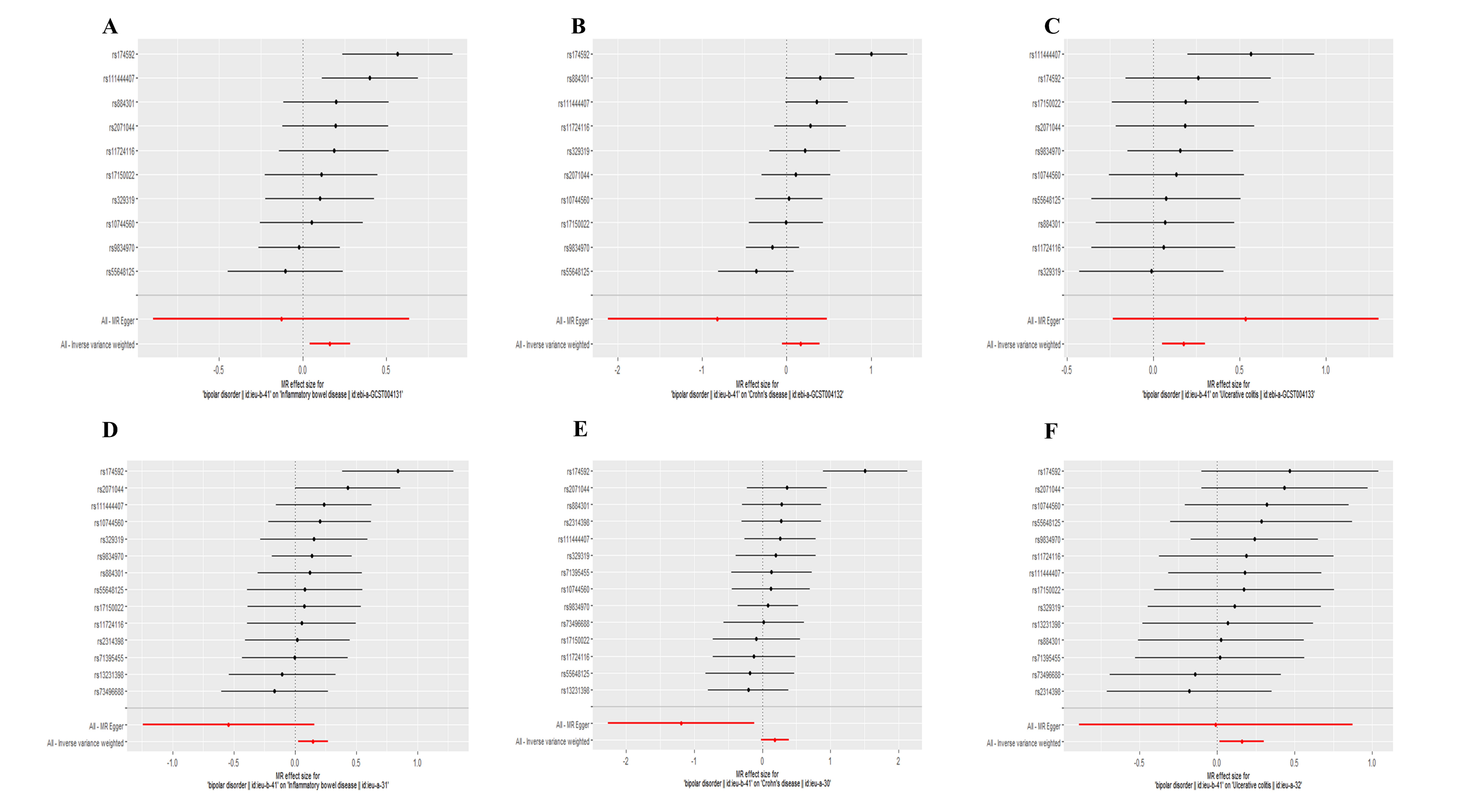

Supplement: Supplementary file 8 [file Image1.TIF]

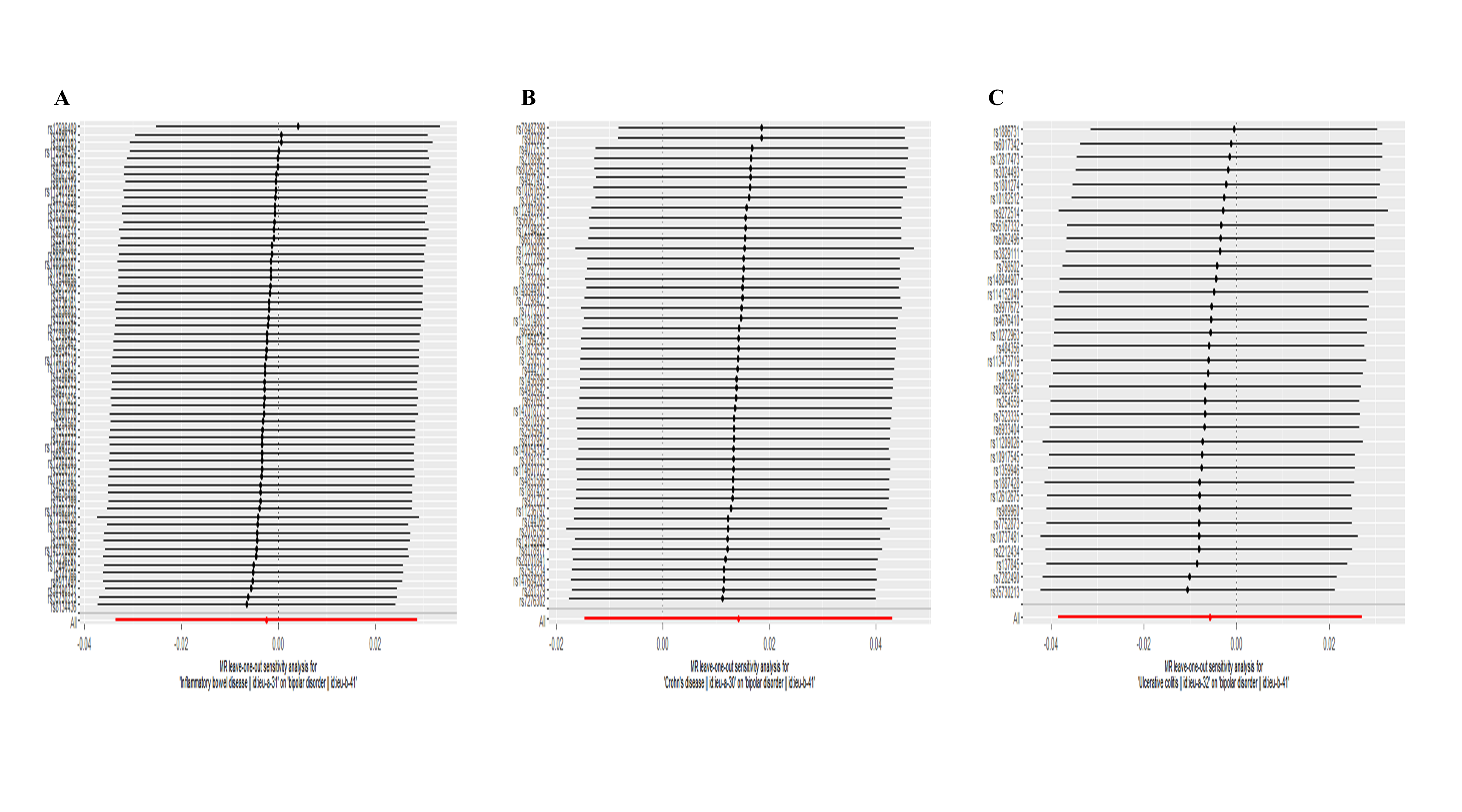

Supplement: Supplementary file 9 [file Image7.TIF]

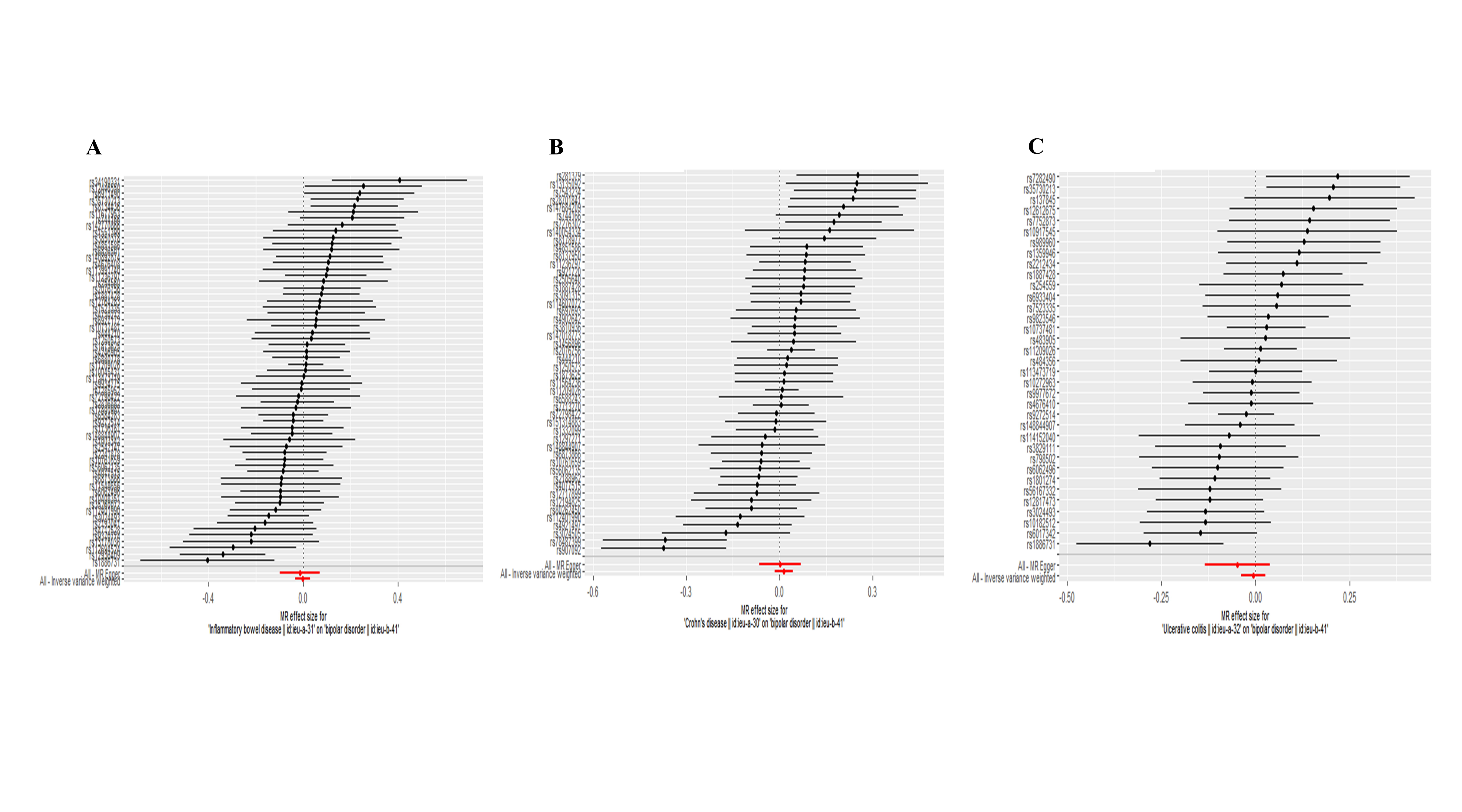

Supplement: Supplementary file 12 [file Image5.TIF]
