# Supplementary material for: Causal relationship between bipolar disorder and inflammatory bowel disease: A bidirectional two-sample mendelian randomization study
Source: Front Genet. 2022 Sep 20;13:970933. doi: 10.3389/fgene.2022.970933 (PMC9531165; doi:10.3389/fgene.2022.970933)
Supplement: Supplementary file 4 [file Table1.DOCX]

Supplementary Materials

**Title: Causal relationship between bipolar disorder and inflammatory bowel disease: A Bidirectional Two-sample Mendelian Randomization Study**

Supplementary Fig 1: Forest diagram of the relationship between genetically predicted BD on IBD, CD and UC. Each horizontal solid line reflects the result estimated by a single SNP using the Wald ratio method and those that cross 0 indicate that the results are not significant. The results of a single SNP may be inaccurate. The red line at the bottom is a combination of the results of the IVW method to obtain reasonable results. (A) BD on IBD*; (B) BD on CD*; (C) BD on UC*; (D) BD on IBD#; (E) BD on CD#; (F) BD on UC#. BD: bipolar disorder, IBD: inflammatory bowel disease, CD: Crohn's disease, UC: ulcerative colitis. * Data from de Lange et al. # Data from Liu et al.

Supplementary Fig 2: Funnel plot of the relationship between genetically predicted BD on IBD, CD and UC. The x-axes represent the effect of instrumental variables on outcomes and y-axes represent standard errors of instrumental variables. (A) BD on IBD*; (B) BD on CD*; (C) BD on UC*; (D) BD on IBD#; (E) BD on CD#; (F) BD on UC#. BD: bipolar disorder, IBD: inflammatory bowel disease, CD: Crohn's disease, UC: ulcerative colitis. * Data from de Lange et al. # Data from Liu et al.

Supplementary Fig 3: Leave-one-out analysis of the relationship between genetically predicted BD on IBD, CD and UC. (A) BD on IBD*; (B) BD on CD*; (C) BD on UC*; (D) BD on IBD#; (E) BD on CD#; (F) BD on UC#. BD: bipolar disorder, IBD: inflammatory bowel disease, CD: Crohn's disease, UC: ulcerative colitis. * Data from de Lange et al. # Data from Liu et al.

Supplementary Fig 4: Scatter plots of the relationship between genetically predicted IBD and its subtypes on BD. (A) IBD on BD; (B) CD on BD; (C) UC on BD. BD: bipolar disorder, IBD: inflammatory bowel disease, CD: Crohn's disease, UC: ulcerative colitis.

Details are the same as in Figure 2.

Supplementary Fig 5: Forest diagram of the relationship between genetically predicted IBD and its subtypes on BD. (A) IBD on BD; (B) CD on BD; (C) UC on BD. BD: bipolar disorder, IBD: inflammatory bowel disease, CD: Crohn's disease, UC: ulcerative colitis.

Details are the same as in Supplementary Fig 1.

Supplementary Fig 6: Funnel plot of the relationship between genetically predicted IBD and its subtypes on BD. (A) IBD on BD; (B) CD on BD; (C) UC on BD. BD: bipolar disorder, IBD: inflammatory bowel disease, CD: Crohn's disease, UC: ulcerative colitis.

Details are the same as in Supplementary Fig 2

Supplementary Fig 7: Leave-one-out analysis of the relationship between genetically predicted IBD and its subtypes on BD. (A) IBD on BD; (B) CD on BD; (C) UC on BD. BD: bipolar disorder, IBD: inflammatory bowel disease, CD: Crohn's disease, UC: ulcerative colitis.

Supplementary Table 1: Instrumental variables for BD

Supplementary Table 2: Instrumental variables for IBD of Liu et al

Supplementary Table 3: Instrumental variables for IBD of de Lange et al

Supplementary Table 4: IBD of de Lange et al on BD

Supplementary Table 5: Association of BD and IBD in MR IVW meta-analyses
